# Learning lessons in emergency management: the 4th International Conference on Healthcare System Preparedness and Response to Emergencies and Disasters

**DOI:** 10.1186/s40696-016-0026-3

**Published:** 2016-10-28

**Authors:** B. Adini, A. Ohana, E. Furman, R. Ringel, Y. Golan, E. Fleshler, U. Keren, S. Reisner

**Affiliations:** 1 Emergency and Disaster Management Division, Ministry of Health, Igal Alon 119, Tel Aviv, Israel; 2 Home Front Command, Ramla, Israel; 3 Rambam Medical Center, Haifa, Israel

**Keywords:** Disasters, Lessons learned, Emergency management, Humanitarian action, Resilience, Training and exercises, Civil–military cooperation, Animal sheltering

## Abstract

**Background:**

The International Preparedness & Response to Emergencies & Disasters (IPRED) conferences are conducted bi-annually in order to share insights and lessons learned from diverse crises. The aim of the article is to bring the IPRED conferences into better professional attention and to share the main insights that were presented in IPRED IV, which was held in January 2016.

**Main body:**

The major lessons learned included: *Planning, regional/global collaboration* and *public*–*private cooperation* should be implemented in developing novel technologies. *International humanitarian action* necessitates coordination between diverse actors concerning all potential threats. *Leadership/coordination* and decision-making capacities of emergency response leaders should be enhanced to ensure quality of care. *Ethics in disaster management*: Triage decisions must not discriminate against terrorists, even when attackers and victims are treated simultaneously. *Resilience management*: Establishing family and community networks increases resilience of individuals and society. *Training programs & exercises* must be evaluated considering cost–benefits. *Human resources*: Teams of experts should be transformed into expert teams. *Communication*: A common disaster-management language needs to be established. Social media is useful due to bi-directional communication. *Civil*–*military cooperation* should be established to facilitate a coordinated response including common terminologies and exercises. *Animal sheltering*: First responders and pet owners are jeopardized if animals are not included in emergency planning. Re-unification of animals with their owners should be included in response models.

**Conclusions:**

IPRED conferences provide a platform for sharing insights and lessons learned from diverse emergencies and disasters. The conferences offer a unique opportunity to share knowledge aimed at improving emergency preparedness, networking between various parties, and substantiates the knowledge and experience of all professionals who take part in the proceedings.

## Background

Disasters strike unexpectedly world-wide and frequently cause significant damage to populations and infrastructure [1, 2]. In the last two decades, numerous countries have had to deal with the tragic consequences of natural or man-made disasters, such as the Haiti and Chile earthquakes (2010), Japan earthquake, tsunami and radiological spill (2011), Nepal earthquake (2015), Philippines Haiyan typhoon (2014), and the ongoing conflict in Syria (2011–present). At the end of 2015 over 21.3 million refugees and 65.3 million internally displaced populations were reported by official United Nations sources, but it is believed that the actual numbers are even higher [3]. More so, terror events are occurring more frequently and in diverse locations, as was seen in the last 2 years in Asia (for example in Afghanistan, Pakistan, Iraq, Israel, Russia), Europe (France, Belgium, Denmark etc.), Africa (such as in Somalia, Sudan, Nigeria), America (for example in the United States), as well as in Australia (Parramatta shooting, 2015). As disasters recognize no borders, practitioners and academicians have acknowledged the need to share insights and lessons learned from managing the diverse crises. This laid the foundation for the International Preparedness & Response to Emergencies & Disasters (IPRED) conferences, which have been conducted bi-annually in Israel since January 2010. IPRED is an international conference directed at sharing lessons learned from emergency preparedness and response to disaster situations; the most recent (fourth) conference was held in January 2016. The conference is a joint initiative, co-organized by the Home Front Command in collaboration with the Israeli Ministry of Health. IPRED IV was attended by over 800 participants, from 34 countries. The majority of the international participants came from the United States (84), China (34), and Germany (20).

The conference’s participants represent a wide variety of organizational affiliations, including ministerial and/or emergency organizations, as well as academic institutions. The partakers consist of both managers and practitioners from many professions, such as physicians, nurses, paramedics, security officers and additional first responders. Civilian and military representatives take part in the proceedings. As the conference was initiated and conducted by military and civilian entities, the planning and execution of all conference’s proceedings was made jointly, while the opening of IPRED IV was launched by the Minister of Health and the Home Front Commander.

The organizing committee is chaired by the head of the Disaster Management Division of the Ministry of Health, the scientific committee is chaired by a well-known expert in disaster management who is both a practitioner and a scientist (the chairman of IPRED IV was formerly the Chief-Surgeon of the Home Front Command), while the chairman of the IPRED conferences is the present Chief-Surgeon of the Home Front Command.

The conference is recognized as a regional convention of the World Association for Disasters and Emergency Medicine (WADEM). It is authorized to accredit 12 Continuing Medical Education (CME) points, granted by the European Accreditation Council for Continuing Medical Education (EACCME).

The aim of the article is to bring the IPRED conferences into better professional attention and to share the main insights that were presented in the recent IPRED IV, which was held in January 2016.

## Scientific program

The conference had three main objectives as follows: (1) to provide a platform for the exchange of experiences and lessons learned for practitioners and researchers involved in preparedness and response to emergencies and disasters; (2) to promote international networking between healthcare professionals; and, (3) to present State of the Art research and enhance international collaboration in studying the fields of emergency and disaster medicine as well as public health preparedness. Considering the wide variety of professional conferences and meetings that are available in the last decade, and in order to attract both scientists and practitioners, the program was designed to engage the participants actively in the proceedings. Overall, 198 presentations in 38 parallel sessions were included in the scientific program, as delineated in Table 1, focusing on topics which invoked a great interest in the past 2 years as well as expansive research. The major themes that were presented in the plenary sessions as well as the different topics that were discussed in the parallel sessions can be found in IPRED IV website (http://ipred4.pwizard.com/wp-content/uploads/web_program.pdf).

**Table 1:** Topics and sessions presented in the IPRED IV conference

No.	Topic	Sessions
1.	Emergency preparedness & response	Controversies in emergency & disaster management Advanced research in disaster management Ethics in emergencies and disasters Lessons learned from recent emergencies and disasters Holistic approach to emergency preparedness Military and civilian collaboration Emerging CBRNe threats Emergency management & sheltering of animals
2.	Emergency medicine	Emergency medicine in mass gatherings Trauma management Trauma management + preparedness in specific circumstances & populations Triage systems and mechanisms during disasters
3.	Managing public health and biological risks	Disease outbreaks and epidemics prevention and control Epidemiology and public health Managing the Ebola crisis
4.	Disaster nursing	The role of nurses in disaster management Emergencies and disaster nursing
5.	Resources	Managing blood resources during emergencies Social networks and volunteering Critical infrastructure resilience: threats, vulnerabilities, cascading effects & interdependencies Utilizing social media in emergency management
6.	Training & education	Medical education and training Simulation tools in triage Emergency medicine education Multi-level exercises: “how-to” session Training Multi-professional capacity building
7.	Logistics	Logistic management of emergencies & disasters Novel technologies in emergency & disaster medicine Technological innovations in emergency management
8.	International humanitarian assistance	International humanitarian action Promoting emergency preparedness by international organizations Humanity in treating refugees & migrants
9.	Planning & Leadership	Models of healthcare emergency planning Quality assurance of disaster preparedness Tools & models in emergency response
10.	Mental health and well-being of individuals & communities	Managing acute stress reactions Promoting population and systems’ resilience

## A large-scale field exercise

**Fig. 1:**
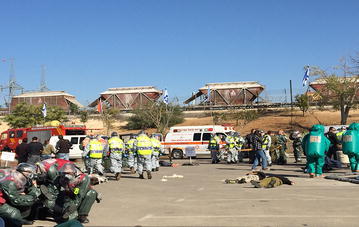
The mass toxicological exercise scene

A unique component of the IPRED IV conference was a large-scale drill which simulated a mass toxicological incident, exemplifying the complexity of such an event, emphasizing the cooperation between the different first responders (see Fig. 1). The goals of the drill were to present the collaboration between civilian and military first responders and rescue entities and display capabilities of multi-disciplinary agencies in the pre-hospital as well as hospital level.

### Lessons identified

The major lessons that were identified as important Take-home messages from the conference’s scientific sessions as well as the mass toxicological drill encompassed the following main elements:

#### Planning and collaboration in emergency management

As emergencies are inevitable, the emergency planning should be founded on an analysis of strengths and gaps and accordingly, mechanisms of collaboration should be developed [2]. Regional and global cooperation need to be established and applied in the development of novel technologies that will promote emergency management [4]. In order to enhance capacities of the various actors involved in the emergency response, public–private cooperation should be encouraged and implemented in the response models [5]. Such cooperation should serve as the basis not only for emergency preparedness and response but also for long term recovery operations.

#### International humanitarian action

As was witnessed in most major disasters that have occurred worldwide in the past decade, numerous international humanitarian entities are involved in the response [6]. Providing an appropriate humanitarian response necessitates coordination between the actors, despite their diverse origins, languages, competencies, and modes of operation. The response needs to be designed in accordance with needs of the particular afflicted population including refugees [7]. Disregard of potential threats and hazards by the public and/or the authorities, must not preclude alertness, as local preparedness is crucial to the establishment and sustainment of a resilient society. The international bodies can significantly enhance local capacities but this can only be achieved if coordination mechanisms are adopted and implemented [8].

#### Leadership/coordination

Global collaboration is a key to successful emergency management [4]. To ensure effective implementation of leadership skills and competencies, decision-making capacities of emergency response leaders should be enhanced. More so, efforts should be invested in the establishment of team coordinators [9], as this facilitates effective operation and ensures quality of care in providing multi-sector, multi-organizational emergency response.

#### Ethics in disaster management

Various ethical issues and dilemmas were identified as relevant to disaster management. A major issue discussed was the impact of a terror act on decision-making concerning triage of casualties [10]. It was stressed that triage decisions must not discriminate against terrorists, despite the highly emotional situation in which attackers and victims are treated simultaneously on-site. Another element that was highly emphasized was that quality assurance of emergency response teams should be based on specified standards [11]. Defining such standards prior to the emergency response will enable to methodologically improve the successful response to different types of emergency scenarios, regardless of their variable components. Another ethical consideration that was raised was the complexity of acquiring informed consent while conducting studies in the realm of emergency/disaster medicine [12]. This is a challenge that should be widely reviewed and weighed in light of the importance for expanding the science of disaster management.

#### Resilience management

Building and maintaining personal, community and national resilience have been extensively studied in the past years. It has been shown that establishing family and community networks increases the resilience of both the individual and the community [1]. Effective resilience management should include not only response capacities, but also take into consideration the need to anticipate risks as well as monitoring their consequences.

#### Training and exercises

Training and exercises have been formerly identified as key components for successful operation and performance during an emergency [13]. Nonetheless, many such activities are carried out lacking evaluation of their specific contribution and effectiveness. The lesson raised in the conference was that training programs, drills and exercises must be accompanied by a strict evaluation and assessment, based on explicit goals and measurable indicators. Though drills are perceived as an important component of emergency preparedness, the type and scope of each drill should be carefully viewed in line of its objectives and cost–benefit should be calculated prior to the conduct of a full-scale drill. Virtual reality as well as simulation training and exercises were found to be an effective alternative to full-scale field exercises [13]. There is great benefit in basing the training and exercises on data from real emergencies as well as on anticipated potential events [11].

#### Human resources

In most mass casualty incidents, numerous individuals, team members and professionals are involved in providing the emergency response [14]. A major goal is to transform a team of experts into an expert team. In order to do so, roles and competencies of all stakeholders must be pre-defined; all professionals have to know what is expected of them, how to carry their roles, and how this will impact other team members and/or the emergency management as a whole [15]. In order to assure the availability of all needed personnel, potential sources of reinforcement must be identified and implemented, such as integration of paramedics as community first care providers or use of veterinary staff as reinforcement of medical personnel.

#### Communication

In order to facilitate an effective flow of communication between first responders and governing authorities that are involved in the response for disasters, a common disaster management language needs to be established [16]. The media is an important tool for informing the population during emergencies and enables stakeholders and actors to effectively communicate during crises. Social media is especially useful as it is based on a bi-directional flow of questions and answers, which facilitates focusing on areas that interest the public [17]. Needs of vulnerable populations, such as deaf or blind individuals, should be integrated in the response mechanisms as an integral part of the risk communication policies [18].

#### Civil–military cooperation

Military systems may provide substantial assistance to civil societies at times of crises. Nonetheless, in order to assure the effectiveness of such a help, the civil–military collaboration should be established prior to the crisis [19]. Common terminologies and guidelines as well as joint exercises must be developed and implemented to facilitate a coordinated response.

#### Animal sheltering

A relatively new area that has been receiving attention from practitioners and scientists studying emergency management is the need to provide an effective response for animal sheltering during an emergency [20, 21]. It has been presented that first responders and pet owners are put at risk if animals are not included in the emergency planning [21]. Though the need to focus attention on this area has been emphasized as especially crucial in agricultural zones, animal sheltering impacts the overall society. Public health and re-unification of animals with their owners should be included in the response model. It has also been stressed that biosecurity is vital, even when animals appear healthy.

#### Managing a toxicological mass-casualty incident

First responders require proficiency in clinical and chemical detection of hazardous materials, to ensure prevention of self-contamination. Treating casualties and communicating between responders while wearing protective gear is challenging and complicate the rescue and evacuation process. A primary isolation perimeter should be marked as soon as a hazmat event is suspected and only forces equipped with protective gear should be allowed access to the scene. A frontal command post should be positioned close to the site of a toxicological event, consisting of representatives from the various first responders. Conducting an After Action Review in which both pre-hospital and hospital staff participate, significantly contributes to identifying lessons.

### Lessons learned from organizing the IPRED IV conference

- The organizing and scientific committees must work as close-knit units in order to achieve the conference’s goals. Appointing key personnel that serve in both committees facilitates efficient networking and flow of information.
- Arranging parallel sessions necessitate careful consideration and should be minimized, as many participants expressed frustration from missing presentations that interest them.
- Some sessions should be planned in different languages (such as French, Spanish or Hebrew), so as to attract more potential participants.
- Utilizing the conference as a platform for facilitating professional meetings of diverse sectors or professional entities should be encouraged.
- Involving a large audience in the drill is challenging and necessitates careful organization and coordination of starting times, to prevent overload of the different sites. It is therefore recommended in future drills to involve more than one hospital so as to enable each participant to observe all aspects of the disaster response without the need to quickly vacate each site in order to allow others to observe the proceedings.

## Conclusions

IPRED conferences have become a tradition and attract many professionals that welcome the platform for sharing insights and lessons learned from diverse types of emergencies and disasters. The present conference was attended by a wide variety of senior representatives from different organizations all over the world. The international experts with vast experience that attended the conference and presented their findings facilitated the diverse educational programs on different aspects relating to emergencies and disasters. The conference offered a unique opportunity to share knowledge aimed at improving emergency preparedness, networking between various parties, and substantiated the knowledge and experience of all professionals who took part in the proceedings. Future IPRED conferences should consider catering to the expectations of additional potential participants by planning some sessions in additional languages as well as designing the mass casualty drill in multiple institutions so as to maximize the exposure of each observer to diverse aspects of emergency management.

## Abbreviations

CME: Continuing Medical Education
EACCME: European Accreditation Council for Continuing Medical Education
EMS: Emergency Medical Services
IPRED: International Preparedness & Response to Emergencies & Disasters
WADEM: World Association of Disaster and Emergency Medicine
